# Cost-effectiveness of a malaria control programs in sub-Saharan Africa: analysis of uncertainties using a stochastic individual-based simulation model

**DOI:** 10.1186/1475-2875-11-S1-P68

**Published:** 2012-10-15

**Authors:** Nicolas Maire, Michael Tarantino, Aurelio Di Pasquale, Melissa Penny, Thomas A Smith

**Affiliations:** 1 Department of Epidemiology and Public Health, Swiss Tropical and Public Health Institute, Socinstrasse 57, P.O. Box, CH-4002 Basel, Switzerland; 2 University of Basel, Petersplatz 1, P.O. Box, CH-4003 Basel, Switzerland

## Background

The first vaccine against *P. falciparum* malaria is close to licensure, having shown moderate levels of protection in clinical trials. Several analyses have shown the vaccine to be likely to be an effective and cost-effective addition to currently used control strategies. We previously used individual-based computer simulations (implemented in the open Malaria simulator) to analyze uncertainties in the predicted cost-effectiveness of introducing a malaria vaccine into the expanded programme on immunization in sub-Saharan Africa. We now extend these studies to include insecticide treated nets, a currently existing intervention. In addition, we also address model uncertainty in the current analysis.

## Materials and methods

We used techniques of probabilistic sensitivity analysis, involving randomly sampling the parameter vectors, to analyze the contributions of the different sources of uncertainty to the predicted cost-effectiveness. One specific aspect of these analyses of uncertainty is quantification of the value of acquiring additional information on these parameters by computation of expected value of perfect information (EVPI).

## Results

Among the most important predictors of the cost-effectiveness of a control program are the cost of the intervention program and the transmission intensity at the time of the start of the program. EVPI is shown to be substantial, and in particular the accrual of up-to-date information on local endemicity would seem an efficient way to inform decisions about local deployment.

## Conclusions

Probabilistic sensitivity analysis and value of information analysis using computer simulation models provide a powerful way to identify data gaps hindering rational resource allocation in malaria control.

